# From Biophysics to Biomedical Physics

**DOI:** 10.1021/acsbiomedchemau.4c00096

**Published:** 2024-12-19

**Authors:** Karim Almahayni, Jana Bachir Salvador, Dijo Moonnukandathil Jospeh, Nazlican Yürekli, Stephanie Möllmert, Leonhard Möckl

**Affiliations:** † 130458 Max Planck Institute for the Science of Light, 91058 Erlangen, Germany; ‡ Department of Physics, Friedrich-Alexander-University Erlangen-Nuremberg, 91054 Erlangen, Germany; § Max-Planck-Zentrum für Physik und Medizin, 91054 Erlangen, Germany; ∥ Department of Medicine 1/CITABLE, Friedrich-Alexander-University Erlangen-Nuremberg, 91054 Erlangen, Germany; ⊥ Deutsches Zentrum Immuntherapie, University Clinic Erlangen, 91054 Erlangen, Germany

**Keywords:** Biophysics, Medicine, Microscopy, Clinical biology, Translational medicine, Super-resolution microscopy, Mechanical phenotyping

## Abstract

The intersection of physics and medicine has long been a fertile ground for innovation, with advances in one field driving breakthroughs in the other. In recent years, the emergence of modern biophysical techniques has provided a toolbox for understanding complex physiological processes across scales. These tools have the potential to revolutionize medical practice, from diagnosis to therapy. However, despite this immense potential, the path from basic biophysical research to clinical translation remains fraught with challenges. This review aims to highlight emerging biophysical tools and their potential applications in medical physics, with a focus on how these innovations can be translated into clinical solutions. We outline a strategic roadmap for bridging the gap between cutting-edge biophysics research and its effective integration into clinical settings, ensuring that these technologies can meaningfully transform patient care.

## Graphical abstract

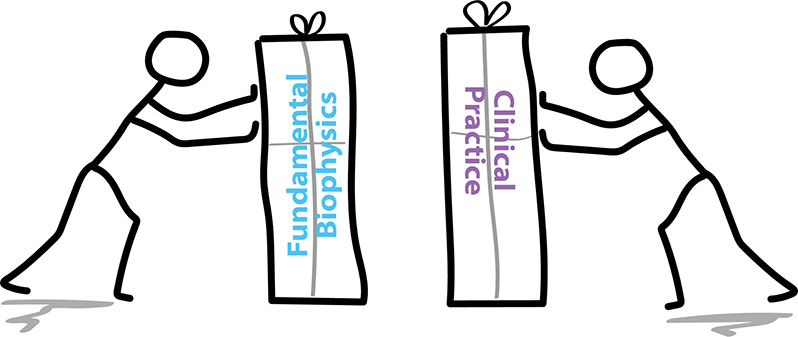

## Introduction

Physics and medicine share a symbiotic relationship, with advances in one field often catalyzing breakthroughs in the other.^1^ Over the past century, medical physics has transformed healthcare, from the development of X-rays and MRI scanners to modern-day radiotherapy and precision imaging techniques.^2^ Recent years have seen a revolution in fundamental biophysical research, enabling the visualization of cellular processes, extraction of mechanical phenotypes and the selective manipulation of biological entities in an unprecedented manner.^3,4^ Despite the immense potential of such tools, the translation of innovative biophysical methods from the lab into clinical practice remains a considerable challenge.^5^ Unlike areas of medical physics that focus on the direct application of physical phenomena in healthcare (e.g., application of radiation phenomena for cancer treatment), recent biophysical research delves into the fundamental understanding of biological processes (e.g., the spatiotemporal dynamics of receptors on the cell membrane and their functional consequences). These recent advances have yet to unfold their full potential in the clinical space; and realizing this vision has enormous potential to improve patient outcome.

This review seeks to address this critical juncture, examining a range of biophysical tools that are on the cusp of clinical relevance. We do not aim for an exhaustive discussion of all techniques available. Rather, we aim to present a perspective of emerging opportunities at the intersection of biophysics and medicine through the lens of a representative sample of methodologies. We believe that the translation of techniques from fundamental research to clinical applications in this field should not be confined to a specialized research domain. Instead, we believe that it will be of tremendous benefit if researchers with no close ties to the clinic are aware of the prospects their methodological arsenal for patient well-being. This is especially the case for techniques that hold significant promise for diagnostics and therapy, but whose direct application is not yet possible due to challenges specific to medical settings.

Among the methodologies we will showcase are advanced imaging techniques, which could surpass traditional clinical imaging modalities in terms of resolution, sensitivity, and target scope. Similarly, developments in mechanobiology and mechano-biotechnology hold the potential to revolutionize personalized medicine, where treatments can be tailored based on the unique biophysical properties of individual patient samples. Beyond surveying the state-of-the-art in biophysics tools, we discuss possibilities of how these innovations could be translated from the laboratory to the clinic, addressing key obstacles and providing a future outlook for method development in this field.

## Advanced Optical Imaging

Advanced optical imaging techniques have considerably improved our understanding of biological systems, providing critical insights into both physiological and pathological processes at the molecular and cellular levels. Optical imaging allows for the visualization of structures and dynamics in living organisms in a fairly noninvasive manner, aiding in disease diagnosis and treatment. For instance, fluorescence microscopy is widely used to observe cellular behavior in real-time, offering valuable information on cellular functions and interactions.^6^ However, when it comes to capturing high-resolution images of subcellular structures or dynamic processes at the nanoscale, more sophisticated tools such as super-resolution microscopy are required, presenting new opportunities and challenges in terms of resolution, speed, and sample preparation.^7^ These advanced techniques push the boundaries of what can be visualized, opening new avenues for understanding complex biological phenomena.^8,9^

This section of the review will focus on super-resolution microscopy techniques, addressing their challenges and pathways for clinical translation. We will also include techniques for single-molecule and -particle tracking, which rely on related physical principles, providing different readouts (extraction of motion information on a target species instead of structural information). A considerable share of advanced optics relies on the power of single-molecule detection and analysis, which has already, in the form of next-generation sequencing, revolutionized diagnostics and therapy.^10^ We think it is not overly optimiztic to expect a second single-molecule revolution in the clinical space within the next two decades.

### What Is “Super” about Super-resolution?

Light microscopy transformed biology in the late 17th century when Antonie van Leeuwenhoek made the first observation of cells using his single-lens system.^11^ Since then, microscopy has continuously evolved, advancing biological research by enabling the study of life’s functional units at increasingly smaller scales. From the single lens microscope system (Leeuwenhoek) to two lens systems (Hooke) to infinity-corrected 3 lens systems (Reichert), the magnification and functionality of the microscope system improved significantly.^12^ Increased magnification, however, is not synonymous with improved resolution. The physical reason for this is the so-called “diffraction limit” as first described by Ernst Abbe and Lord Rayleigh.^13^

Resolution is the ability of an optical system (a microscope in this context) to distinguish two nearby points in a sample. The need for microscopes in the first place arose from the basic limitation of the human eye to resolve two points placed closer than 150 μm. Conventional optical microscopes improved this limit to approximately 250 nm. As described by Abbe, the limit is proportional to the wavelength of the light used to illuminate the specimen and inversely proportional to the numerical aperture of the objective lens.

To understand the resolution a bit more in detail, think of diffraction of light from a point source (object) through a lens. In biological contexts, this would be the fluorescent label attached to e.g. a protein of interest. The resulting diffraction pattern has a spot of maximum intensity and ripples of radially decreasing intensity. This pattern is referred to as the “Airy pattern” or “Airy disc”, which is a good approximation for the Point Spread Function (PSF) of the microscopy, i.e., the response function when imaging a perfect point source.

When two nearby point objects emit light simultaneously, they can be resolved only if they are separated by more than 200 nm. Otherwise, the spot of maximum intensity from the two objects overlaps. Consequently, the diffraction pattern appears as a larger blurred spot with which the information about two emitting spots cannot be obtained. This airy pattern is often called the (lateral; axial also) point spread function of a microscope. Taken together, the diffraction limit applies if two fluorophores emit (i) at the same time and (ii) with the same wavelength. All super-resolution optical microscopy methods circumvent the diffraction limit by removing one of these two conditions, that is, they separate fluorophores either in time or by their emission wavelength (see below).

Toward the end of the 20th century, significant milestones were achieved that lead to overcoming the diffraction limit. In 1989, Moerner et al. detected the first signal from a single molecule.^14^ This laid the groundwork for the development of single-molecule localization microscopy, which was pioneered by Eric Betzig and colleagues.^15–17^ The family of single-molecule localization microscopy methods removes condition (i) from above, separating molecules in time. In the same decade, the work of Stefan Hell established an ingenious way to combine beam shaping and pulsed excitation/depletion to separate fluorophores via emission, removing condition (ii) from above and enabling super-resolution. In recognition of the significant impact super-resolution microscopy had on biological and biomedical research, Moerner, Betzig, and Hell were awarded with the Nobel Prize in Chemistry 2014.^18^

### Single-Molecule Localization Microscopy and DNA-PAINT

Single-molecule localization microscopy (SMLM) is a term used to indicate a collection of techniques for super-resolution relying on the localization of individual molecules. This includes PALM (Photo Activation Localization Microscopy), STORM (Stochastic Optical Reconstruction Microscopy), DNA-PAINT (Point Accumulation of Nanoscale Topography), and others.^15,16,19^ In all of these techniques, emission of fluorophores labeling a structure is tuned in such a way that the emitting molecules are well-separated, i.e., their diffraction limited PSFs do not overlap. A movie with a sparse subset of different molecules emitting in each frame is acquired. Each of these molecules is localized via fitting with an appropriate model function, e.g., a 2D Gaussian. Crucially, this position estimation has a much higher accuracy than the original diffraction-limited PSF on the camera. Thus, by stacking the emitter localization from all frames, one obtains a super-resolved reconstruction of the imaged species (Figure 1).

**Figure 1:**
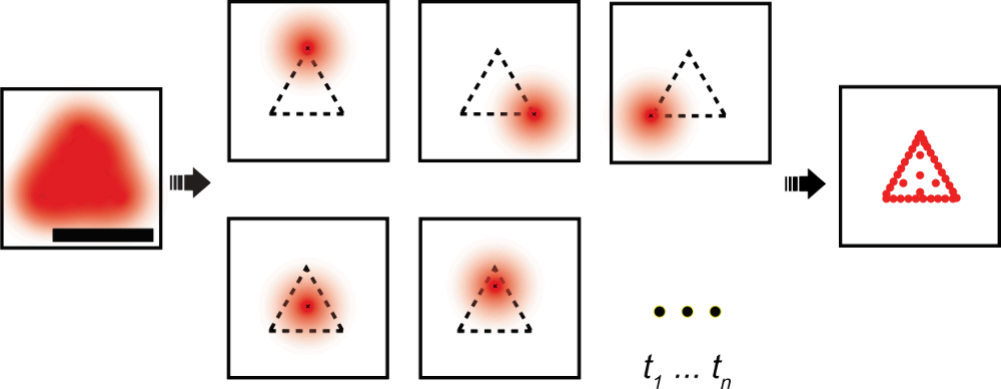
Principle of localization microscopy. If all emitters labeling a structure fluoresce at the same time, all structural detail below the diffraction limit is lost (left). Via separating the fluorophore emission by application of some active control mechanism in time and localizing their centers (middle), a super-resolved reconstruction of the imaged species is obtained (left). Scale bar: 200 nm.

In DNA-PAINT, transient DNA–DNA interactions are employed to yield single-molecule signals (Figure 2A). Two DNA strands are involved in the system. First, the so-called “docking strand”. The docking strand is attached to the imaging target with the method of choice, e.g., via as a primary antibody conjugate. A second DNA strand, called the “imager strand”, is added to the sample and freely diffuses in the medium. The imager strand is complementary to the docking strand and carries a fluorophore, but the emission of this fluorophore is blurred on the camera due to the fast diffusion of the imager strand in the medium. Upon transient interaction between the docking and the imager strand, the imager strand is immobilized, and all photons emitted are originating from one spot, yielding a bright burst of fluorescence. Notably, the whole arsenal of DNA nanotechnology is available to tune docking strands-imager strand interactions, improving specificity, speed, and other relevant parameters.

**Figure 2:**
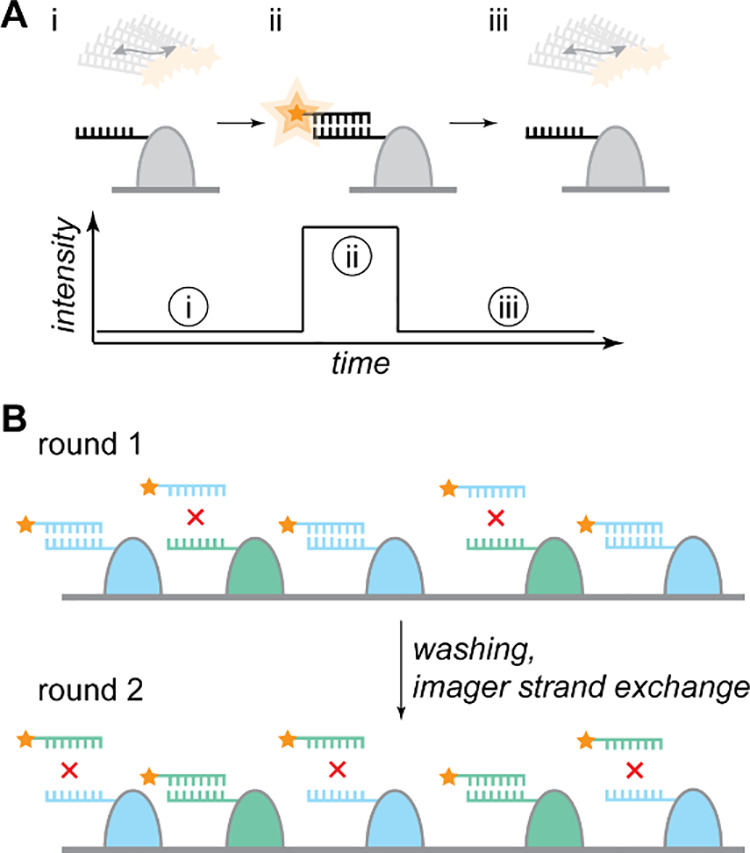
Principle of DNA-PAINT. (A) Transient binding of diffusing imager strands and the complementary docking strands which label the imaging target lead to fluorescent bursts. In the unbound state, rapid diffusion blurs the fluorescence from the fluorophore on the imager strand in the camera frame. (B) Multiplexing via exchange-PAINT. Docking strand 1 is attached to imaging target 1 and docking strand 2 is attached to imaging target 2. The sequences are orthogonal, thus, no affinity between imager strand 1 and docking strand 2 and vice versa is present. Thus, via sequential imaging, the two targets can be visualized. Notably, more targets can be realized by simply introducing more orthogonal DNA sequences to the respective species to be imaged.

In addition, a key advantage of DNA-PAINT is the ability to perform straightforward multiplexing via labeling a large set of imaging targets with orthogonal DNA sequences (Figure 2B).^20^ Due to the reliance of DNA-PAINT onto diffusing DNA strands, this method is particularly straightforward to implement for cytosolic and cell-surface proteins. Nevertheless, imaging targets in more crowded environments have also been realized.^21^ More recently, Secondary label-based Unlimited Multiplexed DNA-PAINT (SUM-PAINT)^22^ and Fluorogenic Labeling in conjunction with Transient Adapter-mediated Switching for High-throughput DNA-PAINT (FLASH-PAINT)^23^ represent two further developments in this field. Both methods address the critical need for highly multiplexed, high-resolution imaging of imaging targets at the molecular scale.

To put the multiplexing capability of these emerging techniques into perspective: Purely optical super-resolution methods are limited in simultaneously imageable targets both in terms of photophysics of fluorophores (not all fluorophores exhibit satisfactory blinking properties) and overlap of emission spectra (if the fluorescent labels of two different imaging targets are overlapping in emission, the unanimous assignment of which emission belongs to which label becomes difficult). In practice, this limits the number of imaging targets to three or four. While this is certainly useful and sufficient in many situations, the recent demonstrations of dozens of imaging targets certainly open up a wholly new perspective on nanoscale imaging of cellular components.^22,23^

Super-resolution microscopy techniques with such multiplexing capability have significant potential for clinical and medical applications. Successful demonstrations of highly multiplexed DNA-PAINT imaging have already facilitated the characterization of membrane receptor distribution at the level of individual receptors. This ability holds significant potential for complex characterization tasks such as molecular imaging of cellular structures. This is due to the addition of nanoscale spatial information as a completely new axis of analysis. With established clinical methods, it is possible to quantify, for example, receptor expression on tissue, which provides a critical clinical metric (e.g., Her2 expression on breast cancer). However, it is conceivable and likely that not merely the expression level, but also the relation of a relevant receptor to other cellular species defines its physiological and pathological function. In order to quickly identify different scenarios of such a regulative network in the clinical setting, a method to reliably provide nanoscale information on multiple species is required.

Thus, clinical implementations of multiplexed super-resolution microscopy could significantly expand the toolbox for cancer subtyping, for example, by adding precise information on spatial organization to established characterization metrics. Synapse biology and neuronal membrane organization has already been demonstrated in a pioneering study,^22^ and it can be expected that improvements in sample preparation, labeling pipelines, and imaging throughput will directly facilitate a deeper understanding of diseases such as autism and Alzheimer’s disease. Another key area where considerable impact of molecular imaging is likely are diseases and treatment strategies related to the immune system. The activity of all immune cells is regulated via a plethora of signaling pathways acting simultaneously, and correspondingly, nanoscale information on the spatial arrangement of regulative proteins has already proven to be of significant impact in fundamental immunology.^24–26^ Standardizing nanoscale analysis of key immune cell receptors in clinical contexts will open up new methods to characterize T cell for cancer immunotherapy, understand principles of pattern recognition in defense against pathogens, and precise analysis of aberrant immune cell state in autoimmune diseases. Notably, as DNA-PAINT inherently relies on DNA–DNA-interactions, it exhibits a natural capacity to be conducted in conjunction with DNA- and RNA-FISH (Fluorescence In Situ Hybridization).^27^ This potential might open up new strategies to perform spatial analysis of gene expression to characterize cancer heterogeneity with single-locus resolution.

Another recent DNA-PAINT-based technology with significant potential for clinical relevance is resolution enhancement by sequential imaging (RESI), a novel super resolution localization microscopy technique that pushes spatial resolution to Ångström scale by collecting multiple localizations for a single target molecule through DNA-barcoded probes imaged sequentially.^28^ Unlike other ultraresolution techniques, RESI uses off-the-shelf hardware and is able to acquire large field of views. This breakthrough enabled the direct observation of dense protein assemblies at the molecular scale.

Ultraresolution methods such as RESI might bring insights to the clinic where molecular relationships at the single-protein level are mandatory. Experimentally, the functional rearrangement of CD20 on the cell membrane upon treatment with the clinically approved monoclonal antibody rituximab has been impressively demonstrated.^28^ Such sensitivity suggests applications in the translational space, where characterization of molecular interactions between small-molecule and their targets could become a new axis in drug development protocols. This capability could furthermore assist in personalized treatments, analyzing the response of individual patients to treatment at the functional level of drug-target interactions.

Such insights might be the first steps toward a general application of super-resolution microscopy in a broad clinical setting: In the booming era of biomarkers, super-resolution microscopy seems to be a central tool considering the molecular dimensions. For the precise detection of biomarkers and to see the correlation between multiple markers, super-resolution microscopy yields the most direct readout, in the natural state of the specimen. Targeted detection of early changes in the structure and distribution of biomarkers in vulnerable populations will help in better therapeutic success compared to a late diagnosis.

Samples collected from incisional biopsy like fine needle aspiration can be used for super-resolution microscopy by remaining close to the physiological state of the specimen. The specimen of interest could also be extracted from blood using currently established extraction/filtration techniques. For samples acquired via surgery, closer observation at the nanoscale can provide detailed information about possible metastatic and migratory pathways, which would be left unnoticed when using other existing diagnostic methods.

An important category of the biomarkers is cellular receptors. However, understanding their role in tumorigenesis is not achieved to the grass root level, such as in the case of estrogen receptors in breast cancer, which have been known for a long time and are used in hormone therapy.^29^ For example, coexistence of progesterone receptors with estrogen receptors have shown better prognosis in hormone therapy.^30^ Super-resolution microscopy has the potential to uncover the fundamental laws governing the role of membrane receptors in cancer via studying their nanoscale organization. Such readouts could then be translated to personalized treatment plans with increased success rates.^31^

To increase throughput and facilitate easy handling, sample preparation for super-resolution microscopy can be automated using a microfluidic system. Furthermore, a microfluidic system with efficient fluid exchange can be integrated with a partially automated imaging system, giving qualitative and quantitative readouts of biomarkers of interests.

### STED and MINFLUX

In contrast to localization-based techniques, which rely on temporal separation of emitters, targeted techniques such as STED (stimulated emission depletion) rely on spectral separation. This is achieved by introducing a second, donut-shaped depletion beam (intensity of zero in the center, Figure 3A). Fluorophores are first brought via a Gaussian-shaped diffraction-limited pulse of laser light into the fist electronically excited state. After a brief delay, which allows excited molecule to relax into the vibronic ground state of the electronically excited state, the STED pulse forces all molecules outside the center of the STED beam to emit fluorescence at the STED wavelength, which is filtered out. Only the molecules in the center of the STED beam, where the STED intensity is zero, can freely fluoresce. These photons are detected. Thus, even though both excitation and STED beam are diffraction-limited, their combination leads to a PSF with an effective size below the diffraction limit, enabling super-resolution imaging of fluorescently labeled target species (Figure 3B).^32^

**Figure 3:**
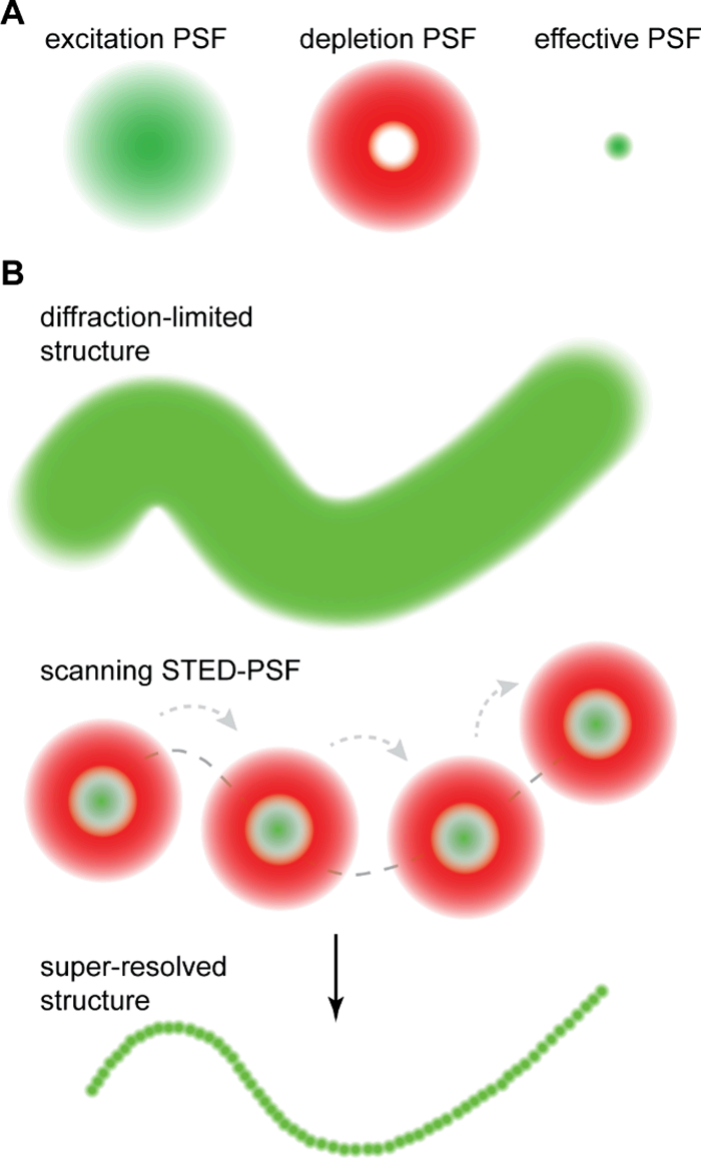
Principle of STED. (A) A Gaussian-shaped excitation beam and a red-shifted, donut-shaped depletion beam are concentrically overlapped. Precisely timed excitation and depletion yields and effective PSF at subdiffraction length scale. (B) Scanning this effective PSF over a fluorescently labeled structure enables subdiffraction limited imaging.

A conceptually related technique that enables even higher resolutions than STED is called MINFLUX (minimal photon fluxes). MINFLUX solely uses a donut-shaped beam, which is scanned over the sample in a tailored manner, quickly zeroing onto the case where the center of the beam perfectly coincides with the position of an individual, well-separated fluorophore. In this case, the photon emission from that molecule is zero. Thus, the position of a molecule is determined by finding the point of minimal photon flux. By iterating this process for all molecules of the sample and recording positions, super resolution of the imaging target is achieved. This procedure can also be used for tracking, where the donut follows a single emitter moving in a biological specimen over time.^33^

In contrast to localization microscopy, STED- and MINFLUX-based methods enable live-cell super-resolution imaging and tracking.^34^ For example, a recent study, tracked the movement of kinesin-1 on microtubules with a spatiotemporal precision of 1.7 nm per millisecond.^35^ Furthermore, in the direction of translating these techniques into clinical use cases, recent reports on deep tissue imaging at a depth of 80 μm with a precision of 5 nm are of particular relevance.^36^

Thus, STED- and MINFLUX-based techniques have already reached a level where their clinical application seems feasible. Expected improvements in labeling of target molecules, reduction in instrumental complexity and ease of handling, as well multiplexing, will further expand the applicability of this family of methods to the clinic. Due to the high achievable penetration depth and the compatibility with live-cell imaging, STED- and MINFLUX-based techniques seem especially powerful in diagnostic imaging of tissue slices or resected tumor tissue at the nanoscale.

### iSCAT Microscopy

iSCAT (Interferometric scattering) microscopy relies on fundamental light-matter information in order to extract position information on scattering objects. It relies on interference of two electrical fields, the scattering field, which originates spherically from a scattering object, and a reference field (Figure 4). Due to the high sensitivity of the interference signal to the position of the scattering object, iSCAT microscopy enables the extraction of nanoscale position information. Furthermore, as the scattering signal is not sensitive to bleaching or other processes that cause loss of signal in fluorescence microscopy, data can be, in principle, obtained without time constraints.

**Figure 4:**
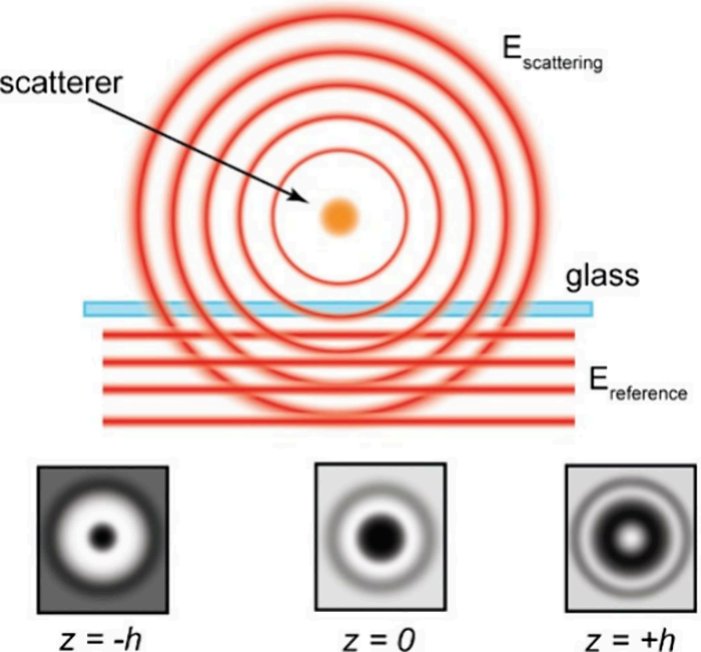
Principle of iSCAT. A scattering object such as a gold nanoparticle gives rise to a spherical scattering field E_scattering_. Interference of the scattering field with a planar reference field E_reference_, which can, for example, generated simply by reflection of the incident field off a glass coverslip, enables highly sensitive position determination of the scattering object in 3D. This is because the iSACT PSF reports with high sensitivity on the position of the scattering object (bottom row: comparison of the iSCAT PSF at three different position of the scattering object around the focus).

Scattering is a universal phenomenon, which enables iSCAT to work without any label. However, for tracking applications, strong scattering is required in order to generate enough signal to reach high spatiotemporal resolutions. In addition, without a label, the target species (e.g., a receptor on the cell membrane) cannot be identified as all other species in the cell scatter as well. Therefore, gold nanoparticles are routinely used due to their high scattering cross section. Using this strategy, nanoscopic motion of epidermal growth factor receptors (EGFRs) on the membrane of Hela cells has been analyzed at microsecond temporal resolution.^37^ Such exquisite spatiotemporal resolutions of protein motion might lend themselves to analysis of drug-mediated functional tuning of membrane dynamics in translational research.

A powerful application of the iSCAT principle is label-free imaging of subcellular structures that exhibit sufficient structural characteristics to be identified by their iSCAT signal alone.^38^ Here, label free live-cell imaging of microtubules, cargo transport inside the cell, endocytosis, and viral dynamics were achieved. This highlights iSCAT as a very promising imaging modality for areas where fluorescence-based methods are not possible. Importantly, as the labeling step becomes obsolete, primary patient-derived material could be analyzed without any intermediate step, which is of particular relevance in clinical settings.

Recently, interferometric scattering mass spectrometry was developed. Here, the iSCAT signal is neither used for tracking nor for imaging. Rather, the relationship between the scattering contrast and the mass of the scattering agent is employed to measure the size of proteins secreted by cells in real time without labeling.^39^ Considering that various processes in health and disease are intimately linked to protein secretion as a means of information exchange between cells, iSCAT mass spectrometry holds considerable promise to diagnostically analyze the secretome of cells or to monitor clinical markers in a noninvasive manner.^40^

### Single Molecule Raman Spectroscopy

While fluorescence microscopy continues to be a workhorse of biological and biomedical studies, it should not be overlooked that by far not all information about a system can be extracted by fluorescence.^41^ One method that holds significant promise is Raman microscopy. In analytical chemistry, Raman spectroscopy is a widely used tool to extract chemical information. Indeed, Raman microscopy combined this exquisite chemical sensitivity with spatial readouts. Bringing Raman microscopy to the single-molecule level would, analogous to single-molecule fluorescence microscopy, greatly enhance spatial resolution while maintaining its significant chemical information content. Despite progress in the field, Raman scattering is, compared to fluorescence, comparably weak, which poses a challenge for single-molecule detection. Besides sophisticated techniques to optically enhance Raman signals, plasmonic enhancements is a promising strategy to increase the sensitivity to the required level.^42^

### What Needs to Happen to Get Super-resolution Methods to the Clinic?

Despite such breakthroughs, super-resolution microscopy still is not where it needs to be for routine applications in clinical settings. In this section, we will examine key obstacles hindering clinical translation of super-resolution microscopy and offer potential strategies for overcoming these barriers.

#### Labeling

In fluorescence imaging, fluorophores attached to proteins are used to detect protein localization. In other words, the position of the fluorophore is taken as a proxy for the position of the imaging target. However, this approach faces challenges in nanoscopy, where the distance between the fluorophore and the protein may exceed the resolution of the experiment, causing a “labelling footprint”. Other issues include incomplete labeling and altered fluorophore photophysics due to crosstalk at low distances.^43,44^ Additionally, large size of antibody pairs (up to 15 nm) can interfere with ultrahigh resolution imaging, not only introducing a labeling footprint, but also preventing labeling of closely packed images targets due to steric hindrance. Smaller probes (e.g., nanobodies and Fab fragments) and direct conjugation (e.g., via click chemistry) may mitigate this issue and offer better reproducibility.

#### Live Cell Imaging and Penetration Depth

Super-resolution microscopy often faces challenges in live-cell applications. Either, data is collected on fixed samples in the first place, such as typically in localization microscopy, or photodamage and photobleaching might turn out problematic. Imaging thicker specimens is challenging due to of out-of-focus background and specimen-induced aberrations. Recent progress in adaptive optics in conjunction with advanced excitation geometries such as light-sheet microscopy holds promise to bring about improvements in this area.^45–47^

Generally, when comparing the two fundamental approaches of super-resolution microscopy (stochastic schemes like DNA-PAINT and targeted schemes like STED), it seems like stochastic schemes are better suited for highly multiplexed, instrumentally fairly simple imaging of large fields of views down to nanometer and even molecular resolution. Unfortunately, live-cell imaging seems currently incompatible with these parameters. On the other hand, targeted schemes provide access to nanoscale information in living systems, but struggle with multiplexing and might face issues related to photodamage.

#### Low Throughput

Compared to conventional imaging modalities, super-resolution microscopy methods generally offer, to date, lower throughput. This is already a challenge in fundamental research, but is arguably even more critical in clinical settings as comprehensive characterization of a specimen is mandatory in order to provide clinicians with a complete picture for, e.g., diagnosis. Advances in robust automation (both in sample preparation as well as data acquisition and analysis) are critical in this area, and recent advances have made impressive strides in this direction.^48^

#### Instrumental Complexity

Once more drawing the comparison to conventional imaging modalities like confocal imaging, super-resolution imaging confronts users with higher instrumental complexity, which also leads to more frequent maintenance. Even the simplest implementations require a comparably high level of expertise for reliable performance and continuous monitoring of quality metrics. Clinical implementations of super-resolution techniques will need to be easier to use and more robust than current laboratory-based solutions.^49–51^ Generally, the goal is not merely to improve patient outcomes, but to democratize access to cutting-edge innovations, ensuring they are effective, affordable, and widely implementable.

#### Data Processing Times

Super-resolution methods usually generate vast amounts of data. Processing gigabytes or terabytes of data can be technically challenging. Integrating existing and future innovations in computational data processing, such as GPU computing, machine learning, tailored image analysis algorithms, and someday perhaps someday even quantum computing will be critical.

In particular, from the methods discussed above, the conceptualization of multiplexed readout is of fundamental importance. Such conceptualization becomes exponentially more challenging with each additional imaged species. Already the mutual relationships between three channels can be demanding for a human, and the generation of data sets with dozens of imaging targets will become routine in the coming years. In other words, we witness the transition from microscopy as a visual tool to microscopy as a data science problem.

A complete multiplexed data set provides a single-shot readout for different targets of interest in a field of view. To compare and draw conclusions from conventional biophysical statistical data analysis such as nearest neighbor distances, it is necessary to adopt another level of perspective in data processing. Recent developments in SMLM data analysis tools for structural classification of localization data is making stronger ties with data science topics. Important tools include Segmentation and Morphological fingerprinting (SEMORE),^52^ Localization Model Fit (LocMoFit),^53^ Automated Structure Analysis Program (ASAP),^54^ and Enhanced Classification of Localized point clouds by Shape Extraction (ECLiPSE).^55^ All of these methods utilize geometric models or shape descriptors, with ECLiPSE using the most number of shape descriptors, to quantify structural characteristics.

With respect to SMLM, a significant and, to date, largely untapped optimization potential is identified at the data recording step. A 16-bit frame of 576 × 576 pixels necessitates approximately 0.6 megabytes of storage. As a consequence of the working principle of SMLM (namely, the recording of well-separated single-molecule signals), the camera frames that are recorded are largely devoid of meaningful information and contain a rather low amount of data. Recently, event-based sensors, which have been utilized in various computer vision applications for years, have been introduced to the single-molecule field.^56,57^ In this approach, no camera frame in the classical sense is recorded. Only when there is a notable change in intensity does the respective pixel of the sensor fire. This allows for the recording of only the on- and off-switching of blinking molecules, while disregarding the multitude of pixels that contain no information, thereby significantly reducing the data size.

## Mechanical Phenotyping

Mechanical properties are increasingly recognized as critical factors for a wide range of biological processes, including cell migration,^58^ differentiation,^59^ and tissue development.^60^ Mechanical properties can serve as key indicators of cellular function, with deviations from normal mechanical behavior often associated with pathological conditions.^61^ Historically, mechanical properties of whole organs serve as biomarkers for disease. For example, palpation is a routine medical examination where a physician assesses the firmness, size, and position of an organ as an indicative measure of its health. More recently, alterations in the mechanical phenotype of individual cells or cell populations have been linked to diseases such as cancer,^62^ fibrosis,^63^ and neurodegenerative disorders.^64,65^ Therefore, studying cellular mechanics as a biophysical parameter, in conjunction with established biochemical or genetic markers, provides a more comprehensive understanding of the underlying mechanisms that govern cellular behavior. This integrated approach not only enhances our knowledge of normal physiological processes but also offers new avenues for early disease detection, diagnosis, and therapeutic intervention.

With such insights integrated in the clinical setting, assessing the mechanical properties of cells and tissue could lead to the development of novel diagnostic tools and personalized treatment strategies, making the study of biological mechanics potentially indispensable in modern biomedicine.

### Atomic Force Microscopy (AFM)

Atomic force microscopy (AFM) is currently considered the gold standard in quantitative mechanobiology. By detecting physical interactions between a modifiable probe and an exposed sample surface, AFM can be utilized to assess elastic, viscous, and adhesive properties of the sample. The key component is a cantilever with a sharp tip or bead at its free end that probes the sample in a piezo-controlled manner (Figure 5A). As the cantilever bends upon interacting with the sample, it causes a change in the path of a laser beam reflected from the cantilever’s surface onto a photodiode. Through calibration, the deflection on the diode becomes indicative of the force *F*(*N*) acting on both the cantilever’s tip, and the sample. As the cantilever approaches the sample and deforms it, a force–indentation curve is generated. By fitting the force–distance curve to specific models, several mechanical parameters can be determined. In the most rudimentary quantification, when using a spherical bead at the cantilever’s tip, the apparent Young’s modulus is typically extracted using the approach segment of the curve where elastic deformation occurs, fitted with a Hertz model modified for a spherical indenter^66^ described by

$$F=\frac{E}{1-\nu^{2}}\left(\frac{a^{2}+r^{2}}{2}\ln\frac{r+a}{r-a}-ar\right)$$

with

$$\delta =\frac{a}{2}\ln\frac{r+a}{r-a}$$

where *F* denotes the indentation force, *E* the Young’s modulus, ν the Poisson’s ratio, δ the indentation depth, *r* the indenter radius, and *a* is the radius of the circular contact area between indenter and sample.

**Figure 5:**
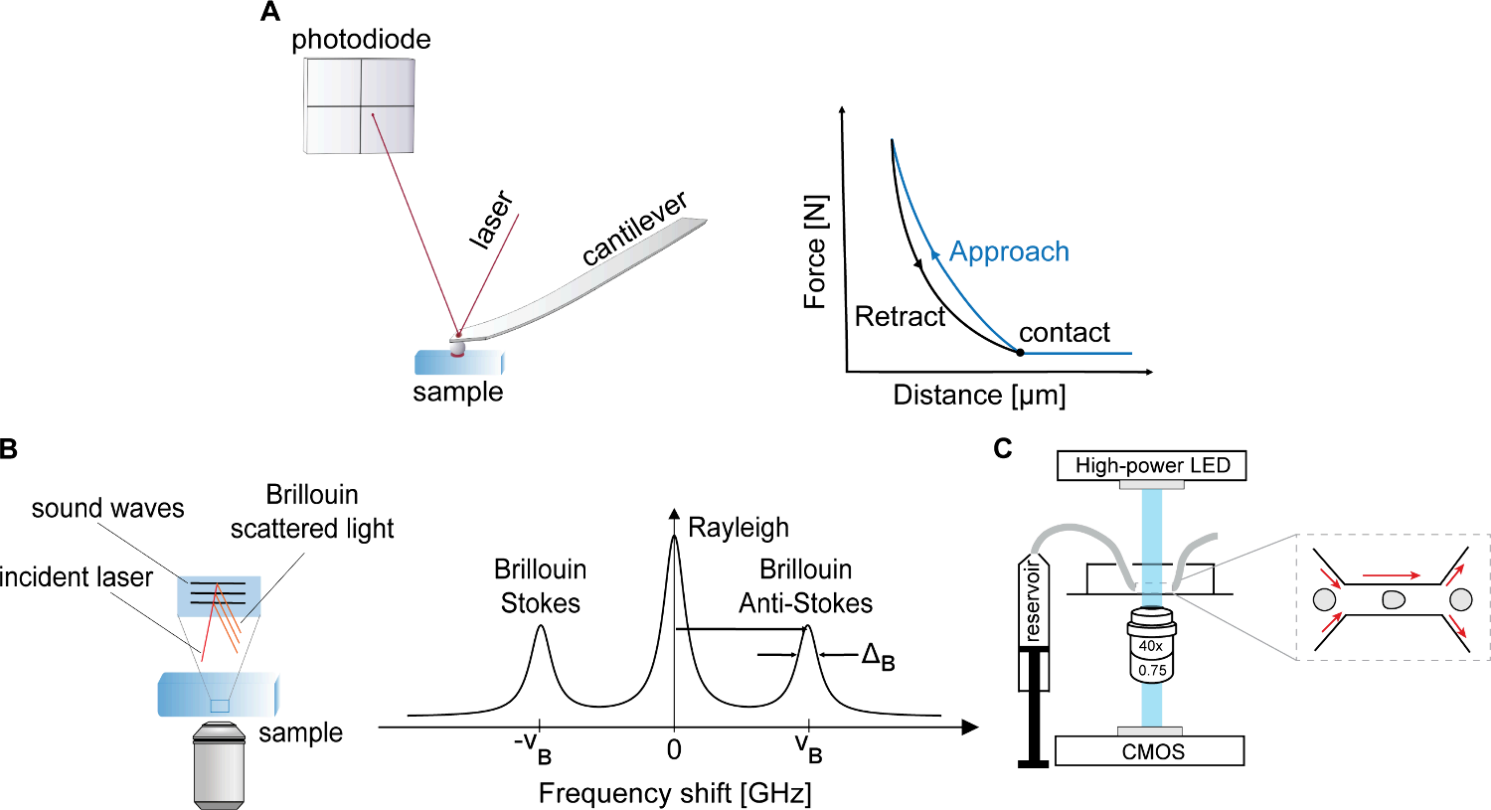
Mechanical phenotyping of cells and tissues. (A) Principle of atomic force microscopy. A cantilever interacts with the investigated sample, and the deflection of the cantilever reports on the local forces between cantilever tip and sample. The cantilever position is measured via a laser being reflected onto a photodiode. (B) Principle of Brillouin microscopy. Light waves interacting with sound waves within the sample report on local mechanical properties. (C) Principle of RT-DC. Cells are pushed through a microfluidic channel. The deformation of the cells within the channel directly reports on their mechanical phenotype.

In the clinical setting, AFM holds significant potential for assessing disease-related cellular and tissue conditions. AFM has been utilized to study 3D microenvironment of tumor spheroid, in order to understand breast cancer progression physiologically.^67^ Furthermore, it highlighted a promising prospect for identifying the nanomechanical signatures associated with distinct stages of tumor progression and metastasis.^68^ The findings revealed that cancer progression involves not only matrix stiffening but also a significant softening of tumor epithelial cells compared to their healthy counterparts. In neurology, AFM has been widely demonstrated to study the mechanical properties of central nervous system tissue and neurons, aiding to identify changes linked to neurodegenerative diseases.^69^ Therefore, by measuring tissue and cellular stiffness, AFM provides nanometer-resolution insights into disease mechanisms and progression, offering potential for diagnostic biomarkers beyond the reach of conventional imaging.

### Brillouin Microscopy

Brillouin microscopy is a noninvasive optical imaging modality that has been adapted to probe the viscoelastic properties of biological cells and tissues. Brillouin microscopy is rooted in a process that is known as Brillouin light scattering (BLS), first reported in the 1920′s.^70–72^ BLS relies on the principle that in all materials above 0 K, traveling acoustic (pressure) waves arise due to thermally generated microscopic density fluctuations. BLS occurs due to interaction of light with these acoustic waves at the gigahertz (GHz) scale. The incident light is scattered by these waves due to local refractive index variations. While the majority of the scattering is elastic (Rayleigh scattering), a small portion is inelastic, known as Brillouin scattering, which involves energy transfer between the light and the acoustic waves, resulting in a measurable frequency shift (Figure 5B).

Brillouin microscopes can be constructed in configurations that give access to several mechanical constants.^73^ Most Brillouin microscopes currently in use, however, primarily probe the longitudinal acoustic waves, which are linked to the longitudinal modulus, *M*, of the material. The longitudinal modulus is a complex quantity that consists of a real component *M*′ (storage modulus) and an imaginary component *M″* (loss modulus):

$$M=M^{\prime }+iM^{\prime \prime }$$

The longitudinal modulus characterizes the material’s viscoelastic behavior, with the storage modulus representing its elastic response and the loss modulus representing its dissipative viscous response. The frequency of the scattered light undergoes both upward (anti-Stokes) and downward (Stokes) shifts. These shifts are symmetric and directly related to the acoustic properties of the material, described by

$$\nu_{B}=\frac{2n\pi }{\lambda }v\;\sin\frac{\theta }{2}$$

whereby ν_*B*_ is the Brillouin frequency shift, *n* is the refractive index of the material, λ is the wavelength of incident light, *v* is the speed of the acoustic wave, and θ is the angle between the incident and scattered light. The speed of the acoustic wave can be inferred from the detected Brillouin frequency shift. This acoustic wave speed is also proportional to the storage modulus *M*′. This means that the Brillouin frequency shift acts as a proxy of the material’s elasticity. Additionally, the line width of the Brillouin peak in the scattering spectrum, proportional to the loss modulus *M*″ provides information about the attenuation of the acoustic waves, which is related to the material’s viscosity and can be expressed by the following relationship:

$$\eta =\frac{\lambda^{2}\rho }{8\pi n^{2}}\Delta_{B}$$

where η is the viscosity of the material, ρ is the density, and Δ_*B*_ is the line-width of the Brillouin peak. Similarly, to the frequency shift, the line-width can then act as a proxy for the material’s viscosity.

By mapping both the Brillouin frequency shift and line width, it is possible to reconstruct spatial maps of the viscoelastic properties of the sample, providing a comprehensive view of the material’s mechanical behavior. Brillouin microscopy has emerged as a powerful and noninvasive technique to visualize and quantify these properties at both the cellular and tissue levels. Recent research underscores its growing potential in medical diagnostics, particularly in detecting pathophysiological conditions characterized by viscoelastic changes.

For instance, in ophthalmology, motion-tracking Brillouin microscopy has been successfully applied to detect keratoconus—an early stage corneal disorder—before it manifests clinically.^74,75^ This innovative technology has already been integrated into clinically viable systems in this field.^76^ Moreover, Brillouin microspectroscopy has demonstrated its utility in distinguishing malignant melanoma from surrounding healthy tissue by assessing differences in Brillouin frequency shifts. Findings revealed that healthy tissue exhibited significantly softer properties compared to nonregressing melanoma, further showcasing the method’s diagnostic capabilities.^77^ These advancements highlight Brillouin microscopy’s potential as a transformative tool for both biomedical research and clinical practice.

### Real-Time Deformability Cytometry (RT-DC)

Despite the undeniable impact of AFM on our understanding of cell and tissue mechanics, its relatively low throughput and technical complexity stimulated the search for techniques that are straightforward to implement with faster readout. This search led to the development of real-time deformability cytometry (RT-DC), which offers a high-throughput alternative that simplifies the process of mechanical characterization by allowing rapid and label-free analysis of mechanical properties of cells.^4^ RT-DC is a microfluidic-based method where cells are suspended in a fluid and flow through a channel. As they pass through a constriction, they are deformed by hydrodynamic forces, without direct contact to the channel wall, and a high-speed camera captures their shape instantaneously. This setup enables real-time measurement of key parameters such as cell size, shape, and deformation. It allows for the analysis of more than 100 cells per second, generating a comprehensive “mechanical fingerprint” of each cell (Figure 5C).^78^

The diagnostic potential of RT-DC has been demonstrated in various pathological conditions, including COVID-19, leukemia, and autoimmune diseases.^79–81^ Recent advancements have extended RT-DC’s application to solid biopsies, where tissue samples are dissociated into single cells, allowing their mechanical properties to be analyzed quickly while keeping the individual cells alive. This innovation transforms the diagnostic process for solid tissue biopsies from a laborious and resource-intensive task into an efficient and automated workflow.^82^

Looking forward, integrating artificial intelligence (AI) with RT-DC holds significant promise. AI could enhance the clinical relevance of RT-DC by correlating physical phenotypes with key diagnostic markers such as tumor malignancy, metastatic potential, and patient survival rates, making it a valuable tool in both diagnostics and clinical trials.

In addition, RT-DC can be engineered to integrate real-time fluorescence imaging (RT-FDC) which enables simultaneous fluorescence and mechanical readouts at the single-cell level.^83^ This capability is crucial for analyzing time-sensitive phases like mitosis, where presorting cells before mechanical measurement would cause delays and prevent accurate phenotyping of such phases. By capturing both fluorescence signals and mechanical properties in real time, RT-FDC retrieves full, comprehensive cellular information, surpassing the limitations of traditional flow cytometry (FCM) or deformability cytometry alone.

It is important to note that while RT-DC, AFM, and Brillouin microscopy assess key mechanical properties, their differing sensitivities across length- and time-scales provide insights into a broad spectrum of relevant material characteristics, spanning multiple orders of magnitude. Although theoretical relationships between the techniques can be established, direct empirical comparisons cannot be applied *a priori*.^84^ Therefore, going forward, the measurement of mechanical properties of biological material will benefit from multiscale and multimodal techniques, with advanced computational models, to allow for a more comprehensive understanding of biological material behavior.

Compared to AFM and Brillouin microscopy, RT-DC generally features higher throughput, which might be beneficial in clinical settings where rapid generation of results on single cells is crucial. On the other hand, AFM and Brillouin microscopy do not rely on individual cells in suspension, but can yield analyses at the tissue and whole organ level, with the potential of *in vivo* applications for diagnostic applications. Thus, RT-DC, AFM, and Brillouin microscopy together form a set of orthogonal techniques that hold significant promise for clinical applications.

## Targeted Cell Manipulation

Cell manipulation and targeting play a pivotal role in both physiological and pathological processes, including tissue regeneration, bacterial inactivation, aging, and cancer treatment.^85–88^ The capacity to precisely manipulate and target specific cells within tissues can markedly enhance therapeutic interventions and diagnostics. Nevertheless, the engineering of processes for targeting individual cells within complex biological environments presents a significant challenge. Over the past decades, several approaches for targeted cell manipulation have been developed and successfully implemented in clinical settings, including microinjection,^89^ nanoparticle-based systems,^90^ and laser microsurgery.^91^

Additionally, several emerging strategies are poised to unlock their full potential in the near future. For example, in cancer therapy, tumors might be selectively targeted by delivering chemotherapeutic agents or genetic materials to them, thus increasing the treatment efficacy and reducing damage to the neighboring tissues.^92^ Similarly, relevant neuron-directed intervention could help treat neurodegenerative diseases like Parkinson’s and Alzheimer’s.^93^ Patient-specific iPS cell lines and motor neurons derived from these cells are crucial for in vitro disease modeling, especially for neurodegenerative diseases like ALS.^94^ In addition, regenerative medicine might also benefit from approaches where stem cells are directed to the damaged tissues for repair.^95^

In particular light is a powerful and versatile tool that can equip targeting with spatiotemporal control. While the earliest ideas of light-mediated therapy can be traced back to ≈3000 B.C.,^96^ recent years have seen a boom in improved technologies, new applications, and new strategies.^97^ To enable targeted and gentle applications, light is usually combined with chemical or genetic probes.

### Optogenetics

Over the past decade, optogenetics has gained significant momentum as a method to manipulate specific cell types and study their effects in real-time *in vivo*.^98^ This technique is especially valuable in neuroscience, where millisecond-scale temporal precision is needed.^99^ Although much of the research has focused on the brain, recent translational efforts have expanded into areas such as vision restoration and cardiac devices.^100–102^ For example, optogenetics has been used to partially restore vision in a blind patient suffering from retinitis pigmentosa, a degenerative retinal disease.^103^ Despite its immense clinical potential, genetic modification of tissues *in situ* remains a challenging task, particularly in hard-to-access tissues such as cardiac muscle.

### Photochemical Approaches

Beyond cell activation, targeted cell ablation is frequently employed in clinical settings to eliminate diseased cells, such as in cancer or age-related conditions, with a high degree of precision and minimal damage to surrounding tissues. Photochemical agents, which are generally simpler to apply than optogenetic methods as they do not require genetic intervention, are commonly used to induce cell death in localized regions. Agents like Photofrin and 5-aminolevulinic acid (ALA) have long been approved for use in early stage and advanced cancers.^104^ However, research into novel photochemical probes faces challenges, including poor water solubility, oxygen dependency, degradation, aggregation, nonspecific tissue distribution, and undesired immunogenicity.^105^ Recent advancements have tackled these obstacles by conjugating photosensitizers with biomolecules to enhance biocompatibility or by formulating them into delivery systems, such as nanoparticles and liposomes. For example, our group recently developed a chemical platform of functionalized bisacylphosphine (BAPO) molecules that offer improved safety, cell permeability, stability, and efficacy.^106,107^ Functionalized BAPOs enable targeted cell ablation in a safe, efficient, and oxygen-independent manner, both *in vitro* and *in vivo.* For example, in a zebrafish embryo-based model system, tissue could selectively be targeted via localized light irradiation, leading to confined cell ablation and neutrophil migration toward the lesion.

Looking ahead, it will be imperative to leverage modern biochemical, optical, and genetic tools to achieve three key objectives: first, to establish disease biomarkers that improve targeting specificity and scope; second, to develop advanced excitation and light delivery techniques for demanding application geometries; and finally, to design cargo delivery systems capable of precisely delivering photochemical and gene-editing probes to defined disease sites.

### Precision Delivery Systems

Although precise delivery in vivo targeting remains challenging in terms of spatiotemporal control, recent studies of *in vitro* delivery systems have demonstrated the ability to confine delivery to the subcellular regime. This concept has been demonstrated with μkiss (microkiss), a micropipette-based technique that applies a layer of small molecules, nanoparticles, or viruses onto the cell membrane from a subfemtoliter flow-confined volume.^108^ Unlike other techniques such as microinjection,^109^ electroporation,^110^ and miniaturized pipettes,^111^ μkiss provides several key advantages: (i) The technique allows for the delivery of materials with micrometer accuracy and millisecond timing, thereby ensuring utmost spatial and temporal precision. (ii) It is versatile, as it operates in an open environment and does not require complex hardware, making it compatible with a wide range of experimental geometries. (iii) It is suitable for live-cell applications, as it is noninvasive and preserves cell viability during the manipulation process.

In therapeutic settings, targeted cell manipulation can be crucial for delivering therapeutic agents directly to diseased cells. μkiss has the potential to perform this task with excellent spatiotemporal accuracy. For example, technologies such as μkiss may prove useful in combating cancerous cells by delivering anticancer agents, such as demecolcine, with precise spatial control, disrupting microtubules and inhibiting further cell division. Similarly, precise delivery of therapeutics will be beneficial in resolving chronic inflammation, for example in Chron’s disease.^112,113^ To ascertain this potential, future studies must assess the in vivo compatibility of μkiss in animal model systems. In diagnostic settings, such as the characterization of biopsies, μkiss has a clear path toward clinically relevant applications. For instance, in virology, single-virus tracking greatly benefits from subcellular precision, allowing functional virus characterization with the required spatiotemporal control.^114–116^ Similarly, resected tissue could be stimulated with therapeutic substances at the single-cell level, which might contribute to facilitating personalized treatment protocols.

## Conclusions—What to Optimize for?

In this Perspective, we have discussed a range of methods that contribute to our ever-increasing knowledge of vital processes in biology and biomedicine. It is our hope that this selection will serve to illustrate the considerable potential of these and related techniques in a clinical setting. However, a recurring theme throughout this Perspective is the significant gap between the theoretical capabilities of these techniques and their practical integration into clinical workflows. While some of the aforementioned methods are more advanced than others, it is reasonable to suggest that all of them will require further development before they can be considered routine tools for clinical use.

It is our conviction that greater attention to the requirements of, and more effective communication with, clinical practitioners is essential to facilitate the transition from fundamental biophysics to medicine; and we believe that a certain shift in perspective is necessary on the part of those engaged in fundamental biophysics. The current research agenda is primarily focused on enhancing parameters that are deemed crucial in fundamental research. For example, in super-resolution microscopy, the relentless pursuit of nanometer-scale precision often comes at the expense of throughput and ease of use, two factors critical for clinical diagnostics. Similarly, advances in mechanical phenotyping tools like atomic force microscopy (AFM) emphasize high-resolution measurements but lack the scalability and speed necessary for routine clinical use.

The proverbial ideal system, for both fundamental and clinical applications, should provide “everything”: It would exhibit ultrahigh sensitivity and throughput, while also demonstrating minimal instrumental complexity, minimal disturbance of the sample, and minimal cost. Nevertheless, this is not a feasible objective. In the pursuit of any gain, one must necessarily make an investment. To illustrate, consider microscopy. The generation of a confocal image of a cell with a resolution of 250 nm requires a specific time and level of complexity on the instrument. Should one desire to enhance the resolution to a level of 15 nm, an investment must be made. Additional measurement time and greater instrumental complexity are required.

Fundamental research seems at times somewhat reluctant to compromise on the parameters that yield the greatest insight into the studied system, such as the maximal resolution of a microscope, “paying” for this optimization via longer measurement times, higher costs, more required user expertise, and lower throughput. However, optimizing solely for maximal insight is not necessarily the optimal metric if a clinical application is desired. To optimize for clinical applicability, a new set of questions must be addressed. To illustrate, let us again turn to microscopy. Assume one is willing to reduce the achievable resolution by 10 nm, which might increase throughput by a factor of 3. An additional reduction of 5 nm in resolution may result in a twofold decrease in instrumental complexity. Is this system still capable to perform the required clinical measurement? If the answer is yes, this system will be a better fit for the clinic than the original design. To answer such optimization questions, a broader and more use case-centered approach is required, which necessitates a detailed understanding of the underlying application scenarios.

To chart a roadmap for translation, future research should (i) foster in-depth dialogue between researchers in fundamental biophysics and clinical practitioners, (ii) accelerate throughput to meet clinical demands, (iii) establish clear metrics of clinical success and streamline pathways for clinical testing, (iv) promote interdisciplinary collaboration across science, engineering, and medicine, and finally (v) enhance accessibility to clinical samples while minimizing bureaucratic barriers.

In conclusion, translating biophysical methods into clinical practice is not merely a technical endeavor; it is a transformative opportunity to reimagine the relationship between science and medicine. We hope that this review will serve as a catalyst, propelling the translation of tools from the lab bench to the patient’s bedside. At the very least, we hope it will spark curiosity on both sides: curiosity of clinicians for innovative, emerging technology in biophysics, and curiosity of researchers in fundamental biophysics for clinical translation.
